# News and Notes

**Published:** 1899-04

**Authors:** 


### NEWS AND NOTES.

DR. W. B. PARKS has removed his office to 201–202 Prudential Building.

DR. W. JAY BELL has removed his office to the Prudential Building.

THE ATLANTA MEDICAL AND SURGICAL JOURNAL has been made the official organ of the Atlanta Society of Medicine. It is an attractive and well-edited monthly journal.—*Maryland Medical Journal.*

DR. CHARLES SHEPPARD, of Macon, Ga., died in that city on March 1 as a result of meningitis.

Dr. M. N. PAULETTE, of Bainbridge, Ga., committed suicide by cutting his throat at his home on March 3.

DR. GEORGE H. STONE, of Savannah, Ga., died in that city on February 19. He was regarded as authority upon yellow fever.

PROFESSOR STRUMPELL, of Leipsic, has just completed his fifty-fifth year of uninterrupted teaching.

THE Birmingham (Ala.) Medical College recently suffered several hundred dollars damage by fire.

AN International Antivaccination Congress is now being arranged to be held in Berlin, June 18, 19, 20.

THE Paris Académie de Médecine is about to build a new home. Reports say that it will be one of the finest buildings in Paris.

---

THE Prince of Wales has accepted the office of President of the National Association for the Prevention of Consumption in England.

---

DR. JAMES H. ETHERIDGE, one of the leading gynecologists in Chicago, recently died in that city. He was professor in the Rush Medical College.

---

DR. FAIRMONT, of Jasper, Ga., was chloroformed and his house robbed on March 8. The burglar was exceedingly daring, and $800 was taken from the house.

---

DR. CHARLES A. L. REED, of Cincinnati, is waging war against the Christian Scientists. He is doing good work in this direction, and should be aided by the entire profession.

---

UNTIL an editor had been elected to succeed the late Dr. John B. Hamilton, Dr. Truman W. Miller, the chairman of the editorial committee, was editing the *Journal of the American Medical Association.*

---

IT has been arranged to have an "International Congress of Ethics" among the congresses of the Paris Exposition in 1900. The organizers are well-known men among the medical profession in France.

---

THE death-rate at Havana for the week ending March 3d was lower than at any time since 1894. During the week there were 187 deaths from all causes. There were no deaths from either yellow fever or smallpox.

Merck & Co., of New York, have acquired all right, title and interest in the *American Medico-Surgical Bulletin.* In its stead will be published *Merck's Archives of Materia Medica and Its Uses.* This arrangement went into effect on the first of the year.

---

A recent medical strike has occurred in the Medical Department of the University of Liege, France. The cause was due to the refusal of admittance to examination of a very hard-working student who had not put in the prescribed number of appearances at lectures.

---

Dr. John Guiteras, in a lecture delivered at the University of Pennsylvania on March 6th, stated his opinion that sixty per cent. of Americans who go to Cuba for the first time will contract yellow fever, and the death-rate among the sick will be from five to six per cent.

---

On March 10th the following statement was issued at Washington showing the total number of deaths reported to the Adjutant-General's office between May 1, 1898, to February 28, 1899: Killed in action, 329; died of wounds, 125; died of disease, 5,277; total, 5,731.

---

The Berlin Medical Society has just held an excited meeting over the admission of women physicians as members. Prof. Virchow, the President, strenuously opposed such action, and the original motion to admit the women was referred back to the committee for further consideration.

---

Dr. E. M. Chamot, of the Chemical Department of Cornell University, states, as a result of a chemical analysis of wall-paper, which he has been carrying on for several months, that nearly all wall-paper sold at the present time contains arsenical poisons, some of them in large quantities.

---

Dr. George B. Fowler, New York city, who was surgeon-in-chief of the army corps commanded by Gen. Fitzhugh Lee, was the guest of honor at a dinner recently given by a number of his professional friends. He has resigned his place with the corps, which is now in Havana, and has returned to his private practice.

---

After so many years of didactic teaching the University of Virginia has decided to add a hospital to its medical department, and an appropriation of $20,000 has been made to inaugurate this movement. The school will also have a course lasting four years from now on, and will once more take its old rank as one of the leading medical schools of this country.

---

President Murphy, of the New York City Board of Health, believing that telephone transmitters are a means of spreading disease, has ordered inspectors to examine the public telephones. The inspectors will thoroughly clease the transmitters and receivers with cotton, which will then be sent to the Bacteriological Department of the Board of Health. Should it be found that telephones are likely to disseminate disease organisms, an order will be issued requiring daily disinfection of the instruments by the telephone companies.

---

A statement compiled in the Adjutant-General's office at Washington shows that the deaths from disease from the first occupation of the camp at Chickamauga in the middle of April to its abandonment in the middle of September, and including the four battalions which remained to January 1st, were 341, or a little less than half of 1 per cent. The deaths of the volunteer force which was mobilized at Camp Thomas up to December 2d, which was two and a half months after they had been withdrawn from Camp Thomas, and after the close of all campaigns in which any of them participated, were for this period of six months 1.61 per cent.

---

DR. MARIE J. MERGLER has been elected Dean of Northwestern University Woman's Medical School, in place of Dr. I. N. Danforth resigned. Dr. Danforth has been elected Dean Emeritus. The yearly course at this school has been changed from one of two semesters to one of four semesters of twelve weeks each, commencing the first of July, October, January and April. Three semesters will be required; the other semester will be optional. The number of regular students will be limited to one hundred—twenty-five in each class. They will be admitted to competitive examination for place in class only after having complied with the requirements of the State Board of Health.

---

DR. EDMUND SOUCHON, of New Orleans, La., President of the Louisiana State Board of Health, and Dr. Quitman Kohnke, President of the New Orleans Board of Health, according to press dispatches of March 13, have been served with bench warrants, commanding that they appear in court at Clinton, La., to answer to the charge of manslaughter before the district court at Clinton. The charge is based upon the alleged concealment of the existence of yellow fever in New Orleans last summer, as through the alleged concealment a citizen of East Feliciana parish contracted the fever in New Orleans and died after returning to his home. The outcome of this will be watched with interest.

---

HUBENERS, in the *German Medical Press*, has reported a series of experiments to ascertain the part played by the mouth of those about the operating-table in producing wound contamination. On an operating-table he disposed crosswise four Petri dishes. Then, having rinsed his mouth with a culture of bacillus prodigiosus, he stationed himself about twenty inches from the nearest plate, and for ten minutes spoke, sometimes in an ordinary voice, sometimes in a low and sometimes in a high one. In every case, and especially when he had spoken in a high voice, cultures of the bacillus developed in the Petri dishes, being particularly abundant in those plates nearest him. Similar experiments conducted by speaking through a mask, similar to an Esmarch chloroform inhaler, containing a layer of absorbent cotton, left the plates sterile.

---

A BILL to prevent premature burial has been introduced in the legislature of New York which provides that every cemetery where the interments are more than 100 annually, shall have a mortuary, well ventilated, the doors unlocked, where bodies can be kept a certain time. The bill also provides that the certificate of the physician must state whether or not all the following signs of death have been noted: 1. Permanent cessation of respiration and circulation. 2. Purple discoloration of the dependent parts of the body. 3. Absence of blistering around a part of the skin touched with a red hot iron. 4. Rigor mortis. 5. Signs of decomposition. No body shall be buried, cremated or otherwise disposed of before twelve hours shall have elapsed from time of death, and before unequivocal signs of decomposition shall have appeared.

---

THE annual meeting of the Western Ophthalmologic and Oto-Laryngologic Association was held in New Orleans February 10 and 11. Owing to the unavoidable absence of the President, Dr. J. Elliott Colburn, of Chicago, the First Vice-President, Dr. W. Scheppegrell, of New Orleans, presided. Two joint sessions and three sessions of the Ophthalmologic and Oto-Laryngologic Sections respectively were held, and many important papers were read and discussed. The following officers were elected for the ensuing year:

Dr. W. Scheppegrell, of New Orleans, President; Dr. M. A. Goldstein, of St. Louis, First Vice-President; Dr. H. V. Wurdemann, of Milwaukee, Second Vice-President; Dr. E. C. Ellett, of Memphis, Tenn., Third Vice-President; Dr. F. C. Ewing, of St. Louis, Secretary; Dr. W. L. Dayton, of Lincoln, Neb., Treasurer. St. Louis was selected for the next annual meeting. The following names were added to the list of honorary members: Dr. Geo. Stevens, of New York; Dr. St. Clair Thompson, of London; Dr. R. Coen, of Vienna, Aust.; Dr. E. J. Moure, of Bordeaux, France; Dr. J. Sondziak, of Warsaw, Russia; Dr. Marcel Natier, of Paris, France; Dr. C. Ziem, of Dantzig, Germany; Dr. A. A. Guye, of Amsterdam, Holland. The new members elected were as follows: Dr. J. A. Caldwell, of McKinney, Texas; Dr. O. Joachin, of New Orleans; Dr. W. H. Baldinger, of Galveston, Texas; Dr. J. S. Nott, of Kansas City, Mo.; Dr. J. S. Lichtenberg, of Kansas City, Mo.; Dr. J. W. Bettengen, of St. Paul, Minn.; Dr. J. W. Chamberlin, of St. Paul, Minn.; Dr. H. M. Starky, of Chicago; Dr. R. Brunson, of Hot Springs, Ark.; Dr. Max Thorner, of Cincinnati; Dr. J. W. Scales, of Pine Bluff, Ark.; E. M. Singleton, of Marshalltown, Iowa; Dr. F. C. Ewing, of St. Louis.

---

PRELIMINARY program of the Georgia Medical Association, Wednesday, April 19th, 10 A.M.

OPENING SESSION.

Address of Welcome, Hon. Emory Speer, Macon.

Response to Address of Welcome, DeSaussure Ford, M.D., Augusta.

Introduction of all the living members present at the organization of the Association in 1849, at Macon, Ga.

1. The Woodbridge Treatment of Typhoid Fever, T. Virgil Hubbard, M.D., Atlanta.

2. Infant Feeding in Health and Disease, Gillham Robinson, M.D., Atlanta.

3. Seven Cases of Diphtheritic Croup—and Treatment, R. M. Harbin, M.D., Rome.

4. The Necessity of a State Pediatric Society, O. B. Bush, M.D., Colquitt.

5. The Endoscopic Treatment of Chronic Urethritis, W. L. Champion, M.D., Atlanta.

6. Some facts in regard to the Medical Complications, Sequella, and Treatment of Typhoid Fever, Hobart A. Hare, M.D., Philadelphia, Penn.

7. (1) Some Fallacies in the Modern Treatment of Nose and Throat Diseases.

8. (2) The Effect Upon the Eye of Electric Flashes of Light, Dunbar Roy, M.D., Atlanta.

9. Technique of: *a*. Suprapubic Cystotomy for Stone; *b*. Varicocele, Willis F. Westmoreland, M.D., Atlanta.

10. (1) Catarrhal Laryngitis.

11. (2) A Suggestion to Increase the Value of Membership in our Association, H. R. Slack, M.D., LaGrange.

12. Mitral Stenosis, M. F. Carson, M.D., Griffin.

13. The Therapeutic Value of Hypnotism, J. R. Rose, M.D., Eastman.

14. The Surgical Treatment of Empyema, W. S. Elkin, M.D., Atlanta.

15. Acute Obstruction of the Bowels, F. W. McRae, M.D., Atlanta.

16. The Best Remedy for Opium Poison, W. A. O'Daniel, M.D. Milledgeville.

17. Mouth Breathing, J. M. Crawford, M.D., Atlanta.

18. Mumps and Metastasis, A. A. Davidson, M.D., Augusta.

19. Nasal Obstruction and Its Influence, J. L. Hiers, M.D., Savannah.

20. An Analysis of Appendicitis Operative Methods, Joseph Price, M.D., Philadelphia, Penn.

21. Appendicitis: From the Standpoint of a Country Doctor, A. C. Davidson, M.D., Sharon.

22. The Importance of a More Thorough Preliminary Education of the Medical Student before Entering College, A. A. Smith, M.D., Hawkinsville.

23. Chronic Diarrhea, T. M. Greenwood, M.D., Mineral Bluff.

24. Emergency Surgery, Nicholas Senn, M.D., Chicago, Ill.

25. A Case of Pachymeningitis, Result of a Chronic Syphilitic Rhinitis, Eugene R. Corson, M.D., Savannah.

26. Report of Cases in Eye, Nose and Throat Practice, Ross P. Cox, M.D., Rome.

27. Malignant Aphthous Tonsillitis or Diptheroid Sore Throat, Mark H. O'Daniel, M.D., Bullards.

28. A Report of Two Interesting Cases, W. D. Travis, M.D., Covington.

29. Improved Perineorraphy, Geo. H. Noble, M.D., Atlanta.

30. The Technique of Rectal Examinations, Jas. P. Tuttle, M.D., New York, N. Y.

31. Some Remarks on the Tact and Delicacy Necessary in Prognosing Ear Cases, Arthur G. Hobbs, M.D., Atlanta.

32. Crawford W. Long and His Discovery, L. H. Jones, M.D., Atlanta.

33. The Physiology of Ether Anesthesia, J. B. Morgan, M.D., Augusta.

34. Ether versus Chloroform, W. H. Elliott, M.D., Savannah.

35. The Administration of Ether and How to Meet its Dangers, S. C. Benedict, M.D., Athens.

36. (Title to be furnished), W. E. Fitch, M.D., Savannah.

37. Adenoid Vegetations in the Post Nasal Region with Especial Reference to their Influence upon the Ear, A. W. Calhoun, M.D., Atlanta.

38. Posterior Displacements of the Uterus, W. B. Gilmer, M.D., Macon.

39. (Title to be furnished), St. J. B. Graham, M.D., Savannah.

40. Some Experiences in a Country Practice, W. L. Story, M.D., Sycamore.

41. The After Treatment of Abdominal Sections, V. H. Taliaferro, M.D., Savannah.

42. The Prevention and Treatment of Pelvic Inflammatory Diseases in the Female by the General Practitioner, R. R. Kime, M.D., Atlanta.

43. The Treatment of Some Cases of Cancer of the Skin, M. B. Hutchins, M.D., Atlanta.

44. A Case of Intestinal Obstruction, from Meckel's Diverticulum, with Gangrene of Intestine—with Specimen, W. P. Nicolson, M.D., Atlanta.

45. Treatment of Carbuncles, Oliver H. Buford, M.D., Cartersville.

46. Asphyxia Neonatorum, Prognosis and Treatment, C. H. Richardson, M.D., Montezuma.

47. Mitral and Aortic Lesions of the Heart—with Specimens, J. L. Campbell, M.D., Atlanta.

48. The Doctor's Scapegoat—the Liver, E. R. Anthony, M.D., Griffin.

49. Puerperal Septicemia, W. N. Edenfield, M.D., Pinehurst.

50. Sympathetic Ophthalmia, Alex W. Stirling, M.D., Atlanta.

51. The Rights and the Wrongs of the Medical Witness in Courts of Justice, Jas. B. Baird, M.D., Atlanta.

52. Surgical Treatment of Varicocele, Wm. S. Goldsmith, M.D., Atlanta.

53. Deaf Mutes with Clinic, M. M. Stapler, M.D., Macon.

OFFICERS FOR 1899.

*President*—Howard J. Williams, M.D., Macon.

*Vice-Presidents*— J. G. Hopkins, M.D., Thomasville; I. H. Goss, M.D., Athens.

*Secretary*—R. H. Taylor, M.D., Griffin.

*Treasurer*—E. C. Goodrich, M.D., Augusta.

*Board of Censors*—Charles Hicks, M.D., Chairman, Dublin; H. B. McMaster, M.D., Waynesboro; W. S. Elkin, M.D., Atlanta; Eugene Foster, M.D., Augusta; S. C. Benedict, M.D., Athens.

*Chairman Committee of Arrangements*—Henry McHatton, M.D., Macon.

*Chairman Committee on Program*—Robert B. Barron, M.D., Macon.

GENERAL INFORMATION.

*Place of Meeting*—Academy of Music.

*Hotels*—Hotel Lanier, $2 and upwards, Mulberry street; Brown House, $2 to $3, Fourth street; Park Hotel, $1.50 to $2, First street; Olympia, $1.50, Second street; Corbett House, $1.50, Poplar street; Stubblefield House, $1, Mulberry street.

*Transportation*—Rates will be four cents for the round trip—three going and one returning. Get certificate from agent at starting point to be signed by the Secretary of the Association before returning.

